# Development of Fluorescence In Situ Hybridization as a Rapid, Accurate Method for Detecting Coliforms in Water Samples

**DOI:** 10.3390/bios11010008

**Published:** 2020-12-24

**Authors:** Jong-Tar Kuo, Li-Li Chang, Chia-Yuan Yen, Teh-Hua Tsai, Yu-Chi Chang, Yu-Tang Huang, Ying-Chien Chung

**Affiliations:** 1 Department of Biological Science and Technology, China University of Science and Technology, Taipei 115, Taiwan; jtk0901@cc.cust.edu.tw (J.-T.K.); chiayuan877@gmail.com (C.-Y.Y.); iambigpighand@gmail.com (Y.-C.C.); hyutang877@gmail.com (Y.-T.H.); 2 Department of Horticulture and Landscape Architecture, National Taiwan University, Taipei 106, Taiwan; flower71625@yahoo.com.tw; 3 Department of Chemical Engineering and Biotechnology, National Taipei University of Technology, Taipei 10608, Taiwan; thtsai@ntut.edu.tw

**Keywords:** biosensor, coliform bacteria, coliform detection method, fluorescence in situ hybridization, water safety

## Abstract

Coliform bacteria are indicators of water quality; however, most detection methods for coliform bacteria are time-consuming and nonspecific. Here, we developed a fluorescence in situ hybridization (FISH) approach to detect four types of coliform bacteria, including *Escherichia coli*, *Klebsiella pneumoniae, Enterobacter aerogenes*, and *Citrobacter freundii*, simultaneously in water samples using specific probes for 16S rRNA. This FISH method was applied to detect coliform bacteria in simulated water and domestic wastewater samples and compared with traditional detection methods (e.g., plate counting, multiple-tube fermentation (MTF) technique, and membrane filter (MF) technique). Optimal FISH conditions for detecting the four types of coliforms were found to be fixation in 3% paraformaldehyde at 4 °C for 2 h and hybridization at 50 °C for 1.5 h. By comparing FISH with plate counting, MTF, MF, and a commercial detection kit, we found that FISH had the shortest detection time and highest accuracy for the identification of coliform bacteria in simulated water and domestic wastewater samples. Moreover, the developed method could simultaneously detect individual species and concentrations of coliform bacteria. Overall, our findings indicated that FISH could be used as a rapid, accurate biosensor system for simultaneously detecting four types of coliform bacteria to ensure water safety.

## 1. Introduction

The levels and types of microorganisms that can exist in tap water are strictly limited by regulatory agencies in various countries. Total coliform bacteria, fecal coliform, and *Escherichia coli* are common indicator bacteria [1]. Coliform bacteria include fecal coliform that originates in feces (e.g., *E. coli*) and not of fecal origin (e.g., *E. aerogenes*). In Europe, fecal *Streptococcus* and *Clostridium* levels are also monitored as indicator bacteria in tap water. Coliform bacteria including *E. coli*, *K. pneumoniae*, *E. aerogenes*, and *C. freundii* have been used extensively as indicators of water quality and are known to affect public health [2]. Thus, determining the levels or species of coliforms is essential for assessing water safety, estimating public health risks, and improving the quality of polluted water. In addition, the detection of coliform bacteria as a main indicator of fecal contamination in water is essential for ensuring water safety. 

The multiple-tube fermentation (MTF) and membrane filter (MF) techniques are commonly used to detect coliforms [3]. MTF can be used to detect the number of coliform groups, i.e., all aerobic and facultative anaerobic, gram-negative bacteria that ferment lactose with gas formation, in water samples. Gas formation in the fermentation tube is a positive indicator for coliforms in analyzed water samples. MF is another method for determining the quality of water samples and involves passing water samples through a vacuum filter. Bacteria present in water samples are filtered through a filter disc and then transferred to a petri dish for bacterial growth. Following incubation, the colonies present on the filter are counted to analyze the bacteria present in water samples. These detection methods use different specific media and incubation conditions to improve detection sensitivity. However, these methods require long detection times, exhibit low specificity, and do not detect nonculturable bacteria [4]. Detection of coliforms based on specific enzymatic activity improves the sensitivity of these methods. The enzyme β-d-galactosidase is widely used for the detection and enumeration of *E. coli* or other related strains [5]. Numerous experiments have shown that this method may be an alternative to traditional *E. coli* detection techniques. However, this detection method is expensive and requires a long incubation time [6,7,8,9]. Molecule-based detection assays, which allow for specific, rapid detection without a cultivation step, have also been proposed for the detection of coliforms. Three molecular-based methods, i.e., immunological approaches, polymerase chain reaction (PCR), and fluorescence in situ hybridization (FISH), have been identified to detect coliform bacteria [10,11,12,13]. 

For immunological approaches, various antibodies against coliform bacteria have been produced, but typically show low antibody specificity, limiting the application of this method [10]. For PCR, various primers have been designed to detect coliform bacteria, e.g., by amplifying the *lacZ* gene (encoding β-galactosidase) for detection of total coliforms and, the *uidA* gene (encoding β-d-glucuronidase) for detection of *E. coli*. Unfortunately, this approach lacks precision and is labor-intensive [14]. PCR/enzyme-linked immunosorbent assay (ELISA) is another good method for detecting coliform bacteria. PCR-ELISA combines PCR and ELISA methods to detect the expression of a selected biomarker. Although our previous studies indicated that PCR/ELISA increased the detection limit and shortened the assay time, this method was found to be affected by the presence of chloride in tap water, resulting in increased error rates [15]. 

In addition, FISH can be used to detect coliform bacteria. FISH uses specific oligonucleotide probes to detect complementary sequences inside cells [16]. The popularity of FISH is related to its advantages of high sensitivity, high stability, cell visualization capacity, safety, short detection time, and multiple color labeling ability [17]. Oligonucleotide probe designed specifically to bind 16S RNA of *E. coli* can be used for microbiological quality control of drinking water samples [18]. Garcia-Armisen et al. [19] combine direct viable counts and FISH to detect viable *E. coli* in river waters and wastewaters. Baudart et al. [20] further used solid-phase cytometry to improve the FISH method for the detection of *E. coli* in seawater, freshwater, and wastewater samples. Barrero-Canosa et al. [21] improved the FISH method by removing the catalyzed amplification reporter deposition steps and combining the hybridization step with fluorochrome-labeled polynucleotide gene probes and rRNA-targeted oligonucleotide probes to shorten the reaction time. Rocha et al. [22] modified a fixation/permeabilization protocol to optimize peptide nucleic acid (PAN)-FISH procedures for detection of *E. coli*. Huang et al. [23] accelerated generic detection of *E. coli* or *K. pneumoniae* in blood culture by combining the PNA-FISH technique and acoustic flow cytometry. Chen et al. [24] and Moffitt et al. [25] have used smFISH to study the expression of the gene NDC80 during meiosis in budding yeast and super-resolution microscopy to image the *Escherichia coli* transcriptome and observe a genome-wide spatial organization of RNA. However, no study used FISH to detect four types of coliform bacteria (i.e., *E. coli*, *K. pneumoniae*, *E. aerogenes*, and *C. freundii*) simultaneously in water samples. 

In the current study, we aimed to develop FISH as a rapid, accurate biosensor system to detect four main types of coliform bacteria, including *E. coli*, *K. pneumoniae*, *E. aerogenes*, and *C. freundii*, simultaneously. We compared the 16S rRNA of these four bacteria and searched for unique sequences of the specific probe to detect these bacteria with FISH. Moreover, we optimized the experimental conditions of FISH for detecting these bacteria. Our findings provided insights into the use of FISH for fast detecting coliform bacteria in water samples.

## 2. Materials and Methods

### 2.1. Bacterial Strains

The strains of coliforms were kindly provided by the Bioresource Collection and Research Center (BCRC) of Taiwan and included *E. coli* strain ATCC23815 (serial no. BCRC 10314), *K. pneumoniae* ATCC9997 (serial no. BCRC 11644), *E. aerogenes* ATCC13048 (serial no. BCRC 10370), and *C. freundii* ATCC8090 (serial no. BCRC 12291). 

### 2.2. Probe Design

We used the BLAST function on the NCBI website to determine the presence of the specific sequences in the 16S rRNA of the four coliforms. These specific sequences were also confirmed by BLAST function to be unique to these coliform bacteria and not existing in other coliform bacteria in water samples. Moreover, the total cell number detected using FISH was similar to that identified using the plate counting method. This indicates the absence of cross-reaction in these bacteria. These probes, which contained the sequences GAGTAAAGTTAATACCTTTGCTC (16S rRNA target sequence), CGGTGAGGTTAATAACCTCTCGA (16S rRNA target sequence), GCGAGTAACGTCAATCGCCAAG (16S rRNA target sequence), and AAGGCGTTGTGGTTAATAAC (16S rRNA target sequence), were used in FISH to identify strains of *E. coli*, *K. pneumoniae*, *E. aerogenes*, and *C. freundii*, respectively. Cy3 (550 nm excitation, 570 nm emission), FAM (494 nm excitation, 519 nm emission), Texas red (595 nm excitation, 613 nm emission), and Cy5 (650 nm excitation, 670 nm emission) were used to label the 5′ ends of the probes to detect these four bacteria, respectively. We also compared these probes with other bacterial databases of 16S rRNA sequences and found that these probes were specific for the four coliform strains.

### 2.3. Culture of Coliform Bacteria and Pretreatment of Samples 

Pure cultures of coliform strains were grown in Luria Broth (Thermo Fisher Scientific, Waltham, MA, USA) and prepared according to the manufacturer’s instructions. Briefly, 200 mL of culture was placed in a 500-mL flask and grown at 37 °C (with shaking at 150 rpm) for 7–8 h. Aliquots of 20 mL of culture broth were centrifuged at 7500× *g* for 5 min at 4 °C. After the supernatant was removed, the pellets of coliform bacterial cells were washed with 10 mL of 1× phosphate-buffered saline (PBS: 130 mM NaCl, 10 mM Na_2_HPO_4_, 10 mM NaH_2_PO_4_, pH 7.2) and resuspended in 30 mL of 3–4% (*w*/*v*) paraformaldehyde or 2–4% ethanol in 1× PBS to identify the optimal conditions for fixative components. The suspension was mixed and fixed for different times (1, 1.5, or 2 h at 4 °C or 25 °C) to determine the optimal fixation conditions. The solutions were centrifuged at 13,000× *g* for 2 min, and the pellets were collected and resuspended in 1× PBS. Next, 10-μL droplets of the pellet suspensions were applied to glass slides and air-dried at ambient temperature. The slides were dehydrated in 50%, 75%, and 96% (*v*/*v*) ethanol for 2 min and air-dried again.

### 2.4. FISH Conditions

For analysis of FISH conditions, 16 μL hybridization buffer with 1 µL stock solution for the probe (50 ng/µL) was placed onto pellets of coliform bacterial cells on glass slides. Slides were exposed to different hybridization temperatures (42, 46, 50, or 62 °C) for different hybridization times (30 min to 3 h) on a rotating incubator. The negative controls consisted of hybridization buffer without the addition of coliform cells and probes or coliform cells (or river water samples) without probes subjected to standard FISH conditions. We prepared a stock hybridization mix by adding 250 µL of formamide, 100 µL of 10× SSCP and 100 µL of 50% dextran sulfate to 20 µL of 10 mg/mL sonicated salmon sperm DNA. We heated the hybridization mix to 70 °C. For each pair of wells to be hybridized, we added 9.4 µL of hybridization mix to 1 µL of probe DNA and mixed thoroughly. After hybridization, the slides were washed with washing buffer at different temperatures (44, 48, 52, or 64 °C) for 20 min. The slides were further rinsed with distilled water and air-dried. 

### 2.5. Epifluorescence Microscopy

Microscopy was conducted using an epifluorescence microscope (Leica DMLB, Germany) equipped with a 50-W power supply, mercury lamp, several filter sets, and a camera (CoolSNAP Pro; Media Cybernetics, Inc., Rockville, MD, USA). For detection of coliforms with our specific probes, a narrow-range CY3 filter was used to detect Cy3; FAM green filter was used to detect FAM; a Tex red filter was used to detect Tex Red; a Cy5 filter was used to detect Cy5. For cells stained with DAPI (359 nm excitation, 461 nm emission), a UV filter was used. Samples were examined using a 1000× oil immersion lens or a 400× dry objective lens. Images were analyzed with Image-Pro Plus Version 4.5 (Media Cybernetics, Inc., Rockville, MD, USA). For the identification of coliforms, the following criteria were considered: (i) bright signal in the CY filter channel emitted by the cells; (ii) positive staining of the cells for DAPI, FAM, Cy3, Texas red, and Cy5; (iii) no emission in other channels when Cy3 was not emitting light (to distinguish false positives from autofluorescence); and (iv) cell morphology resembling that of coliform bacteria. The number of counted viewing fields was 20–100. For the total bacterial count, all DAPI-stained cells (blue) were counted in randomly chosen microscopic viewing fields delineated by the eyepiece micrometer.

### 2.6. Detection of Coliform Bacteria in Simulated Water and Domestic Wastewater Samples

In order to evaluate the sensitivity and environmental applications of this FISH method, we analyzed coliform bacteria in the simulated water and domestic wastewater samples. There were many interference factors present in the water; thus, we used simulated water samples to analyze the feasibility of the FISH method. Tap water was autoclaved and mixed with the designed concentrations of bacteria (2.8 × 10^7^ CFU/mL for *E. coli*, 2.2 × 10^7^ CFU/mL for *K. pneumoniae*, 2.6 × 10^7^ CFU/mL for *E. aerogenes*, and 2.4 × 10^7^ CFU/mL for *C. freundii*). We prepared four glass slides with bacterial pellets from water samples. These four glass slides were incubated with four different probes (i.e., specific probes for the four types of coliform bacteria). Using our established FISH method, we determined the cell numbers of these four coliform bacteria in simulated water samples. In addition, other traditional methods (coliform detection kit, MTF, MF) were also used for comparison with FISH. Our FISH biosensor system was also used to detect these four coliform strains in actual domestic wastewater (10 L collected from the Taipei Wastewater Treatment Plant in Taiwan). The collected wastewater samples were centrifuged for analysis. Prior to the analysis, we performed three individual experiments to identify the background concentrations of the general bacterial population. All analyses were conducted in triplicate, and the mean values have been listed in Table 1 in the revised manuscript. 

## 3. Results

### 3.1. Establishment of the Optimal Fixation Conditions for Detecting Four Types of Coliform Bacteria Simultaneously

**Figure 1:**
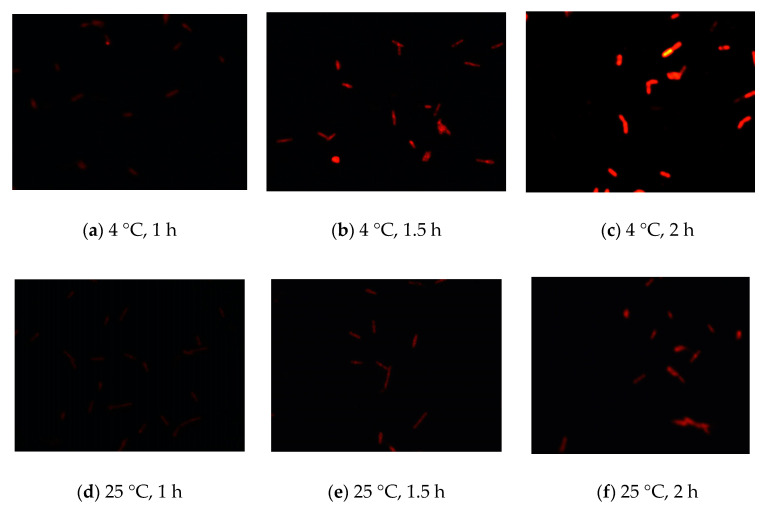
Establishment of the optimal fixation conditions for detecting *E. coli* using fluorescence in situ hybridization (FISH). (**a**–**c**) *E. coli* cells were fixed using 3% paraformaldehyde at 4 °C for different times (1, 1.5, or 2 h) and then stained with Cy3. (**d**–**f**) *E. coli* cells were fixed using 3% paraformaldehyde at 25 °C for different times (1, 1.5, or 2 h).

To develop FISH as a rapid method for detecting coliforms, we first established the optimal fixation conditions for FISH. We used the BLAST function of the National Center for Biotechnology Information (NCBI) to find the 16S rRNA of these bacteria and design unique sequences for the specific probe. Moreover, we optimized the experimental conditions for fixation compounds. The different concentrations of fixation compounds, e.g., ethanol (2–4%) or paraformaldehyde (3–4%), were used to fix cells, and Cy3 was then used to stain cells. The results showed that fixation with 2–4% ethanol was ineffective and 3% paraformaldehyde was the optimal fixation compound for detection of *E. coli* using FISH by judging the intensity and sensitivity of fluorescence. Similar results were obtained for other coliform bacteria, including *K. pneumoniae*, *E. aerogenes*, and *C. freundii*. In addition, we also compared the fixation performance at different temperatures (4 °C and 25 °C) and times (1, 1.5, and 2 h). Based on the intensity and sensitivity of fluorescence, the results showed that the optimal fixation temperature and time were 4 °C and 2 h, respectively (Figure 1). These experimental conditions were also optimal for staining with 4′,6-diamidino-2-phenylindole (DAPI), fluorescein (FAM), Texas red, and Cy5.

### 3.2. Identification of the Optimal Hybridization Temperature and Time for Detecting Four Types of Coliform Bacteria Simultaneously

To identify the optimal hybridization temperature and time for specific probes to detect four coliform bacteria, we tested different hybridization temperatures (42, 46, 50, and 62 °C) and hybridization times (0.5, 1, 1.5, 2, 2.5, and 3 h). We used Cy3, FAM, Texas red, and Cy5 as dyes and applied specific probes at different hybridization temperatures for 1.5 h to detect *E. coli*, *K. pneumoniae*, *E. aerogenes*, and *C. freundii*. The intensity of the fluorescence signal in bacteria and the background noise signal were used as indicators for evaluating the optimal FISH conditions. 

**Figure 2:**
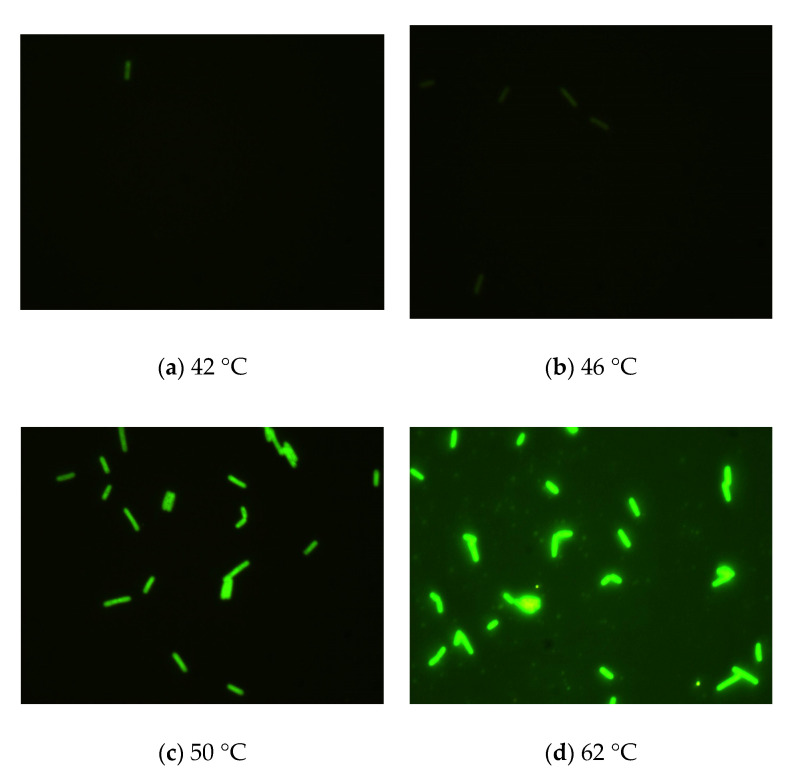
Identification of the optimal hybridization temperature for detecting *K. pneumoniae* using FISH. (**a**–**d**) *K. pneumoniae* cells were detected by FISH with specific probes at different hybridization temperatures (**a**: 42 °C, **b**: 46 °C, **c**: 50 °C, and **d**: 62 °C) for 1.5 h.

**Figure 3:**
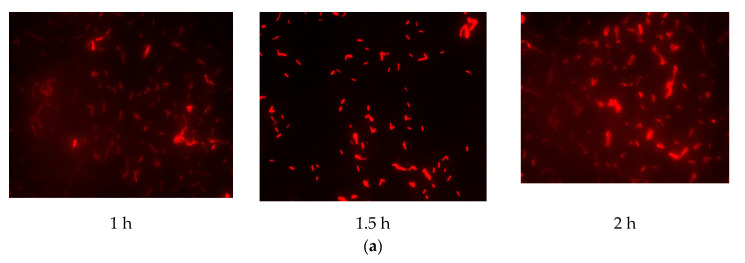

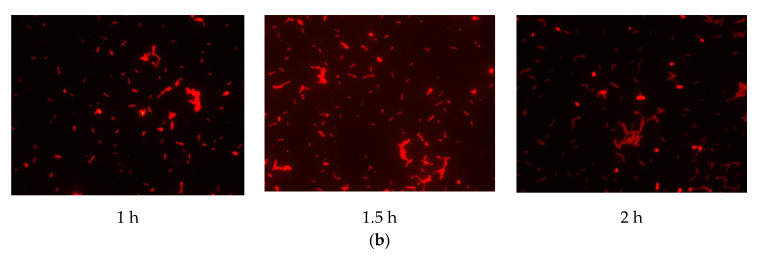
Identification of the optimal hybridization time for detecting coliform bacteria using FISH. *E. coli* (**a**) and *E. aerogenes* (**b**) cells were detected by FISH using specific probes for hybridization for different times at 50 °C.

**Figure 4:**
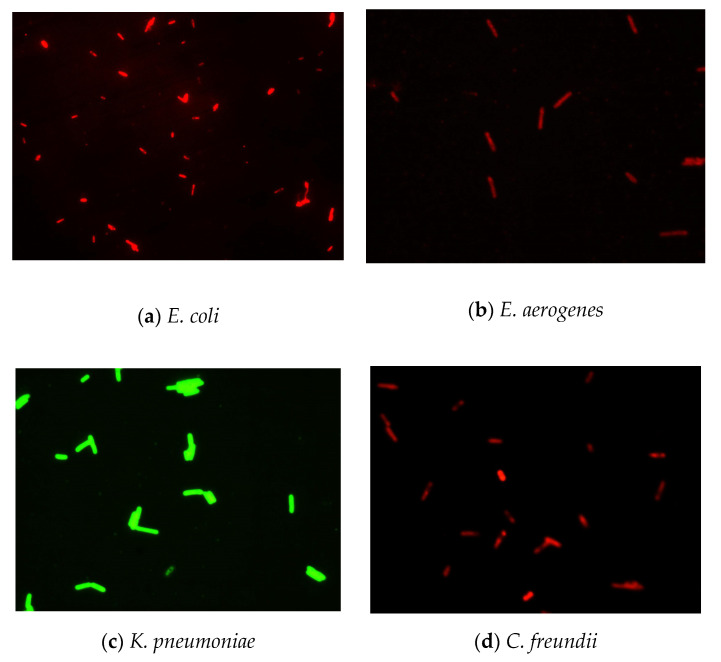
Detection of four coliform strains using the established FISH method under optimal conditions.

**Figure 5:**
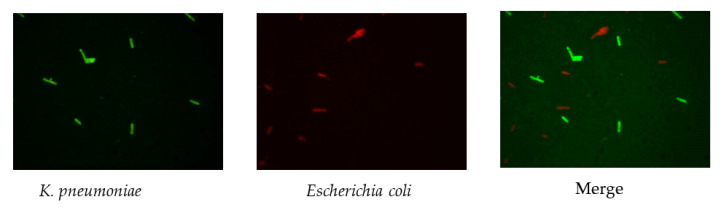
Simultaneous detection of *E. coli* and *K. pneumoniae* to demonstrate the specificity of the probes in FISH.

The results (Figure 2) indicated that there was a pronounced fluorescence signal for *K. pneumoniae* when using specific probes at hybridization temperatures of 50 °C or 62 °C, whereas a distinct noise signal was observed at the hybridization temperature of 62 °C. Thus, the optimal hybridization temperature for detecting *K. pneumoniae* using specific probes in FISH was 50 °C (Figure 2). Similar results were obtained for the other three coliform strains, i.e., *E. coli*, *E. aerogenes*, and *C. freundii*. Further analysis revealed that the optimal hybridization time was 1.5 h for detection of *E. coli* (Figure 3a), *E. aerogenes* (Figure 3b), *K. pneumoniae*, and *C. freundii*. At other hybridization times, the fluorescence intensity was lower or the noise intensity was increased. Moreover, the optimal exposure times when using Cy3, FAM, Texas red, and Cy5 were 1.5, 4, 2, and 8 s, respectively. Finally, we used the optimal FISH conditions for detecting these four types of coliform bacteria, i.e., *E. coli*, *E. aerogenes*, *K. pneumoniae*, and *C. freundii* (Figure 4). The number of DAPI-stained bacterial cells represented the total bacterial count. By overlapping these FISH figures under the same field of view, the cell number of target strain would be estimated, and simultaneous detection of *E. coli* and *K. pneumoniae* to demonstrate the specificity of the probes in FISH (Figure 5). The results showed that the developed FISH approach was effective for the specific detection of these coliforms.

### 3.3. Comparison of FISH Detection with Traditional Coliform Detection Methods for Coliforms Detection in Simulated Water and Domestic Wastewater Samples

To determine the sensitivity and reliability of FISH for detecting coliform bacteria, we prepared simulated water samples containing *E. coli*, *K. pneumoniae*, *E. aerogenes*, and *C. freundii*. We used spectrophotometric analysis and plate counting to confirm the exact cell numbers of these bacteria. Moreover, we mixed each coliform strain at concentrations of 2.5 ± 0.3 × 10^7^ CFU/mL in simulated water and then detected these bacteria using FISH under the optimal experimental conditions identified above or with traditional coliform detection methods (i.e., plate counting, MTF, and MF).

Furthermore, we also compared the total detection times and accuracy of FISH with those of traditional coliform detection methods. The results showed that MTF requires the longest detection time (4 d) and the error rate of the analysis is relatively high. In contrast, FISH has the shortest detection time (4 h) for the identification of coliforms in the simulated water samples, and the total cell number detected by FISH was similar to that of the plate counting method (Table 1). These results suggested that FISH may be the best detection method for identifying these four coliform bacteria in water samples.

**Figure 6:**
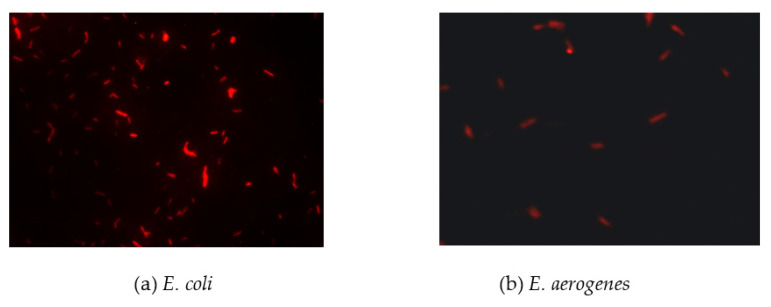
Detection of coliform bacteria in domestic wastewater using FISH.

To evaluate the feasibility of FISH for coliform detection in real environmental water samples, we obtained domestic wastewater from Taipei Wastewater Treatment Plant (Taiwan). In this study, we used a commercial coliform detection kit instead of plate counting to accurately detect bacterial numbers of total coliform. We also used optimal FISH conditions for detecting these four coliforms and compared them with the results from standard methods, i.e., MTF and MF, and a commercial coliform detection kit (Merck, Darmstadt, Germany). We further compared the accuracies of FISH, MTF, MF, and the commercial coliform detection kit. The results of domestic wastewater analysis were similar to the results of simulated water samples (Table 1). The FISH result has been provided in Figure 6. FISH also had the shortest detection time among the tested methods, and the total cell number (4.9 × 10^6^ CFU/mL) was similar to that (4.8 × 10^6^ CFU/mL) obtained using traditional detection methods (e.g., MF) for detecting coliforms in domestic wastewater. Notably, the FISH method can simultaneously distinguish the composition of coliforms as *E. coli* and *E. aerogenes*. In a comparison of the accuracies of FISH and the commercial detection kit, we found that FISH was superior at detection time (Table 1).

## 4. Discussion

In the current study, we established FISH as a rapid, accurate method for detecting fecal (*E. coli*, *K. pneumoniae*) and nonfecal (*E. aerogenes*, *C. freundii*) coliform bacteria simultaneously. We first designed specific probes based on the unique 16S rRNA sequences of these bacteria. Moreover, we optimized the experimental conditions for FISH, including fixation components, fixation temperature and time, hybridization temperature and time, and dye detection time, to detect these four coliform bacteria. For application, we use the developed FISH method to detect the four coliform bacteria in simulated water samples and domestic wastewater samples and compared the results with those from traditional coliform detection methods. Our results demonstrated that the FISH approach established in this study was the best detection method with the shortest detection time and relatively low error rate for detecting fecal and nonfecal coliform bacteria simultaneously in water samples and domestic wastewater samples.

Table 2 lists the performance parameters of conventional methods and FISH methods. MTF and MF are traditional methods commonly used to detect *E. coli* or coliform bacteria [3]. However, these methods require long incubation times and long preparation times and have low sensitivity and specificity; thus, several molecular detection methods, i.e., immunological approaches, PCR, PCR/ELISA, real-time loop-mediated isothermal amplification, indirection competition ELISA, FISH, and PNA-FISH have been developed to overcome these limitations [19,20,21,22,23,24,25,26,27,28,29,30]. Among these detection methods, FISH has been shown to be an extremely useful diagnostic tool for the detection of coliforms or other bacteria based on its rapid turnaround time and high accuracy [19,20,26,27,28]. In FISH, specific probes are vital for the detection of microorganisms. In previous studies, specific probes were designed to detect three different pathogenic bacteria, i.e., *E. coli*, *E. faecalis*, and *Staphylococcus aureus*. Probes Eco541 and Eco1482 were identified by Fuchs et al. [31] and Tang et al. [32] to detect *E. coli*. In contrast, probe Eub338 was shown to recognize all bacteria broadly [28], and two other probes (Ecoli and Colinsitu) were designed using 16S rRNA for detecting *E. coli* [33,34,35]. In this study, we identified specific probes to detect four coliform bacteria, i.e., *E. coli*, *K. pneumoniae*, *E. aerogenes*, and *C. freundii*, using FISH. The probes were designed for specific detection based on the conserved 16S rRNA sequences of the bacteria using the BLAST function of NCBI. These specific sequences were also confirmed using the BLAST function to be unique for these coliform bacteria and did not exist in other coliform bacteria found in water samples. Therefore, the probes identified in this study were used for the first time in FISH and could detect the four bacteria simultaneously. The standard protocol for FISH involves hybridization at a temperature of 46 °C for 2–3 h and uses the intensity of the fluorescence signal to evaluate the performance of FISH [31,36,37]. Several studies have indicated that hybridization of DNA probes at lower temperatures for longer times can preserve the morphology of the targeted chromosome and yield higher fluorescence intensities. For example, Winkler et al. [38] applied hybridization at 37 °C for 15 h. Buno et al. [39] also used a low temperature (37 °C) for hybridization for 4–5 h. Tang et al. [32] demonstrated that the probe Eco1482 had lower fluorescence intensity but higher hybridization efficiency compared with Eco541 when performing FISH at 46 °C for 3 h or 55 °C for 30 min. Low-stringency hybridization (i.e., low temperature) corresponds to stronger binding of probes to the targeted rRNA sites [31]. 

These findings indicate that higher temperatures may be less suitable for FISH in terms of obtaining a satisfactory fluorescence signal. In this study, we found that the optimal hybridization temperature for the specific probes during FISH was 50 °C and that the optimal hybridization time was 1.5 h for detecting the coliforms evaluated in this study. We further evaluated whether the differences between the optimal temperatures of our probes and those of other published probes, e.g., Eco541 and Eco1482, may be related to the probe design using prediction software. However, the predicted temperatures of these probes were different from their optimal temperature as demonstrated by FISH experiments. Therefore, the different probes had optimal hybridization conditions, and these differences may not be related to the probe design.

Several studies have applied FISH to detect *E. coli* and coliform bacteria in water samples. For example, Garcia-Armisen et al. [19] and Baudart et al. [20] used an improved FISH method to detect viable *E. coli* in river water, wastewater, seawater, and freshwater samples. In addition, Ootsubo et al. [40] used FISH with probe D following cultivation for enumeration of Enterobacteriaceae in food and environmental water samples, and the experimental time was within 7 h. Hügler et al. [41] also developed and validated a FISH-based method for the detection and quantification of *E. coli* and coliform bacteria in water samples. Their protocol consisted of two approaches: direct detection of single *E. coli* and coliform bacteria on filter membranes, and incubation of the filter membranes on nutrient agar plates with subsequent detection of microcolonies. Huang et al. [23] detected *E. coli* and *K. pneumoniae* in 5 h in blood culture using the PNA-FISH-AFC technique. However, these methods require long culture times or expensive equipment and can only be used to detect a single *E. coli* strain. In addition, fecal and nonfecal coliforms were not separately detected, and no studies had evaluated the levels of coliform bacteria from domestic wastewater using FISH. 

In our study, we used our established FISH method to detect four coliform strains (*E. coli*, *K. pneumoniae*, *E. aerogenes*, and *C. freundii*) in water samples and compared the results with the results from other common detection methods, i.e., plate counting, MTF, or MF. We first used spectrophotometric analysis and plate counting to develop simulated water containing four coliform strains, i.e., *E. coli*, *K. pneumoniae*, *E. aerogenes*, and *C. freundii*, at a concentration of 2.5 ± 0.3 × 10^7^ CFU/mL. Thus, we were able to know the exact number of each coliform in the simulated water, and this could help us to determine the accuracies of the methods. Accordingly, we compared the detection times and accuracies of these methods to demonstrate that the novel FISH assay enabled short detection times and yielded low error rates. Moreover, we used domestic wastewater samples to evaluate the detection times and accuracies of these methods for detecting coliforms in real environmental samples. We showed that our FISH system was superior to the other methods in terms of short detection time and low error rates. Taken together, these results applied FISH as a rapid, accurate biosensor system for detecting coliform bacteria in water samples and domestic wastewater samples.

## 5. Conclusions

In summary, we identified four specific probes for detecting four main types of coliform bacteria, i.e., *E. coli*, *K. pneumonia*, *E. aerogenes*, and *C. freundii*. We further used these four probes to establish optimal FISH conditions to detect these four types of coliform bacteria simultaneously. By comparing the established FISH with the common detection methods, we demonstrated that the FISH method had a shorter detection time (4 h) than traditional methods (1–4 d) and high accuracy for detecting coliforms in simulated water samples and domestic wastewater samples. In addition, FISH could be used to detect cell numbers of each of these four coliform strains and directly observe the cell bodies of these bacteria; our results revealed that FISH had high sensitivity for identifying the four species of coliform bacteria and suggested that this FISH method may have potential applications for detecting or identifying fecal and nonfecal coliforms simultaneously in water samples.

## Figures and Tables

**Table 1:** Comparison of FISH with other traditional methods for detecting coliform bacteria in simulated water samples or domestic wastewater samples.

Simulated Water Samples						
	Detection Time	*E. coli*	*K. pneumoniae*	*E. aerogenes*	*C. freundii*	Total Cell Numbers (CFU/mL)
Plate Counting *	24 h	2.80 ± 0.31 × 10^7^	2.20 ± 0.30 × 10^7^	2.60 ± 0.33 × 10^7^	2.40 ± 0.32 × 10^7^	1.00 ± 0.21 × 10^8^
Multiple-Tube Fermentation (MTF)	4 d	−	−	−	−	8.00 ± 0.43 × 10^8^
Membrane Filter (MF)	24 h	−	−	−	−	2.60 ± 0.23 × 10^8^
FISH	4 h	4.30 ± 0.13 × 10^7^	4.10 ± 0.16 × 10^7^	3.20 ± 0.25 × 10^7^	2.80 ± 0.21 × 10^7^	1.44 ± 0.14 × 10^8^
**Domestic Wastewater Samples**						
	**Detection Time**	***E. coli***	***K. pneumoniae***	***E. aerogenes***	***C. freundii***	**Total Cell Numbers** **(CFU/mL)**
Coliform Detection Kit (Merck)	24 h	−	−	−	−	2.60 ± 0.21 × 10^7^
Multiple-Tube Fermentation (MTF)	4 d	−	−	−	−	8.30 ± 0.38 × 10^6^
Membrane Filter (MF)	24 h	−	−	−	−	4.80 ± 0.33 × 10^6^
FISH	4 h	3.80 ± 0.22 × 10^6^	−	1.10 ± 0.15 × 10^6^	−	4.90 ± 0.36 × 10^6^

* Using a standard coliform strain and normal culture conditions for these bacteria on NA agar plates at 37 °C. −: not detected.

**Table 2:** Comparison between the FISH method and conventional water detection methods.

Detection Method	Detection Time	Operating Procedures	Distinguish the four Coliform Groups	Analysis Cost	Accuracy
Plate Counting	Medium	Simple	Yes	Cheap	-
Coliform Detection Kit	Medium	Simple	No	Medium	Medium
Multiple-Tube Fermentation	Long	Not Complicated	No	Cheap	Low
Membrane Filter	Medium	Not Complicated	No	Cheap	Medium
FISH	Short (4 h)	Complicated	Yes	Medium	High
